# Postoperative radiotherapy improves survival in completely resected non-small cell lung cancer with pathologic N2 stage IIIA and positive lymph node count greater than one: a SEER-based retrospective cohort study

**DOI:** 10.3389/fsurg.2024.1506854

**Published:** 2025-02-04

**Authors:** Diyang Zhu, Yuanyuan Xiao, Shancheng He, Baochang Xie, Wenqi Zhao, Yuhui Xu

**Affiliations:** ^1^ Department of Internal Medicine, The Second People’s Hospital of Yudu County, Ganzhou City, Jiangxi Province, China; ^2^ Department of Critical Care Medicine, Ganzhou Fifth People’s Hospital, Ganzhou, China; ^3^ Department of Critical Care Medicine, Ganzhou Respiratory Disease Control Institute, Ganzhou, China; ^4^ Department of Pulmonary and Critical Care Medicine, Ganzhou People’s Hospital, Ganzhou, Jiangxi, China

**Keywords:** non-small cell lung cancer, postoperative radiotherapy, overall survival, positive lymph nodes, stage IIIA pathologic N2

## Abstract

**Objective:**

Non-small cell lung cancer (NSCLC) constitutes approximately 85% of lung cancer cases, with 20%–30% of patients diagnosed at stage III. While multimodal therapy is the standard for treating locally advanced NSCLC, the role of PORT remains controversial. This study seeks to evaluate the effect of postoperative radiotherapy (PORT) on overall survival (OS) and cancer-specific survival (CSS) in patients with resected pathologic N2 (pN2) stage IIIA NSCLC.

**Methods:**

Data from the Surveillance, Epidemiology, and End Results Program (SEER) 17 registry (2010–2019) were analyzed. The cohort included 1,471 patients aged 65 years or older, diagnosed with stage IIIA pN2 NSCLC, who had undergone lobectomy or total pneumonectomy. Patients who had received neoadjuvant chemotherapy or radiotherapy were excluded. Univariate and multivariate analyses were conducted to assess the association of PORT with OS and CSS. Kaplan-Meier survival curves were employed to estimate survival outcomes, while the COX proportional hazards model was utilized for comparative analysis. PLN counts were stratified into two categories: ≤1 and >1.

**Results:**

Among the 1,471 patients included in the study, 613 (41.67%) received PORT, while 858 (58.33%) did not. PORT was associated with a significantly higher 1- and 3-year OS (89.96% and 68.49%, respectively) compared to the non-PORT group (87.44% and 61.88%, respectively, *P* = 0.03). However, no significant difference in CSS was observed between the groups (*P* = 0.15). Among patients with PLN counts >1, PORT significantly improved OS (HR = 1.32, 95% CI = 1.04–1.68, *P* = 0.0016) and CSS (HR = 1.32, 95% CI = 0.99–1.70, *P* = 0.026), whereas no significant differences were seen in patients with PLN counts ≤1.

**Conclusions:**

This study underscores the potential of PORT in enhancing OS in patients with resectable pN2 stage IIIA NSCLC, particularly in those with PLN counts exceeding one. These findings suggest that PORT may offer improved outcomes in patients with extensive lymph node involvement, emphasizing the need for further prospective studies to validate and expand upon these observations.

## 1 Introduction

Lung cancer remains a predominant contributor to cancer-related mortality. Non-small cell lung cancer (NSCLC) constitutes approximately 85% of all cases, with 20%–30% of patients presenting with stage III disease at diagnosis (1). For those with locally advanced (LA) NSCLC (stage IIIA-C), multimodal therapy is the cornerstone of treatment (2–4). Individuals with stage IIIN2 NSCLC are usually treated with concurrent or sequential chemoradiotherapy (CRT), and some may get immunotherapy (5). This is especially true for patients with unresectable cancer (IIIA4, IIIB). Alternatively, a surgical approach, involving either double or triple modality treatment, may be employed for patients with resectable stage III NSCLC (IIIA3) (6). A very heterogeneous cohort, stage III pN2 NSCLC exhibits a variety of clinicopathological parameters, including the size of the original tumour, the number of afflicted stations or areas, the volume of the lymph nodes (big vs. non-large), and the histological subtype. The survival advantage conferred by chemotherapy in patients with fully resected NSCLC has been substantiated through numerous phase III trials and meta-analyses, which report an absolute survival benefit of 5% at 5 years (7). Postoperative Radiotherapy (PORT) refers to radiotherapy administered after surgical resection to improve locoregional control and potentially enhance survival outcomes in patients with NSCLC. For patients with stage IIB to IIIA disease, neoadjuvant or adjuvant chemotherapy is recommended. Postoperative radiation, or PORT, is frequently recommended for patients with stage III N2 NSCLC; nonetheless, there is still continuing discussion over its usefulness. Thus, we conducted an analysis of data from NSCLC patients within the N2 population, using the latest SEER database, to elucidate the role of PORT in pN2 NSCLC and explore the relationship betweenpositive lymph nodes (PLN) count and the use of PORT.

## 2 Methods

### 2.1 Data source

We utilized data from the most recent Surveillance, Epidemiology, and End Results Program (SEER) 17 registry database (submitted in November 2021), which encompasses records from 2010 to 2019. The SEER database is a publicly available and widely recognized data source, representing approximately 26.5% of the U.S. population. Its standardized data collection and comprehensive coverage make it an invaluable tool for population-based cancer research. This database integrates data from SEER 17 registries, including San Francisco-Oakland, Connecticut, Detroit, Hawaii, Iowa, New Mexico, Seattle-Puget Sound, Atlanta, the Alaska Native Registry, Georgia (excluding Atlanta), Kentucky, Louisiana (New Orleans), New Jersey, the Great Plains (spanning Iowa, Kansas, Minnesota, and parts of Nebraska), California (excluding San Francisco-Oakland and Los Angeles), Utah, and Los Angeles, as cataloged within the SEER17 database. Collectively, SEER17 represents approximately 26.5% of the U.S. population, based on the 2020 Census. This study received ethical approval from the Ganzhou Fifth People's Hospital.

### 2.2 Cohort selection and outcome

We employed SEER*Stat version 8.4.3 (seer.cancer.gov/seerstat) to compile the case cohort. Patients included were those diagnosed with stage IIIA non-small cell lung cancer, pathologic N2 classification, no distant metastases, aged over 65, who had undergone lobectomy or total pneumonectomy, and had at least one lymph node evaluated. All patients were classified as having resectable pN2, defined as pathologic N2 disease deemed resectable based on preoperative clinical evaluation and completely removed via surgery. However, due to the limitations of the SEER database, further distinction between preoperatively known N2 and intraoperative incidental N2 could not be made. Similarly, the database does not provide details on single-station vs. multi-station N2 involvement. Patients who received neoadjuvant chemotherapy or radiotherapy were excluded, along with those with missing tumor characteristics, pathologic details, or follow-up information. Ultimately, 1,471 patients were incorporated into the analysis cohort. The following variables were included: age, race, gender, year of diagnosis, primary tumor site, histologic grade, T-stage, and surgical approach. Race was classified as white, black, or other. The primary outcome is overall survival (OS), while the secondary outcome is cancer-specific survival (CSS).

### 2.3 Statistical analysis

Univariate analysis was conducted to assess the relationship between each confounding factor and OS or CSS. A COX proportional hazards model was employed to compare survival rates between the PORT group and the non-PORT group. The Kaplan-Meier method was utilized to estimate OS and CSS. The x-tile software was used to define the threshold for PLN count, which was determined to be 1, thus categorizing the PLN variable into two groups: ≤1 and >1. In the COX model, PLN stratification was performed to evaluate the prognostic significance of PORT within these two groups. Patients were stratified based on the number of positive lymph nodes (PLN ≤1 vs. PLN >1) to evaluate the prognostic impact of PORT. This classification was used as a proxy to assess the extent of lymph node involvement, given the lack of detailed station-level data in the SEER database. All statistical analyses were performed using Empower (R) (X&Y Solutions, Inc., Boston, MA, USA) and R version 3.6.3. offers robust data processing and comprehensive analytical capabilities. Statistical significance was set at *P* < 0.05.

## 3 Results

### 3.1 Baseline characteristics of study participants

A total of 1,471 patients were included in the study cohort. Of these, 613 (41.67%) received PORT, while 858 (58.33%) did not. The proportion of male participants was 43.4%, and female participants constituted 56.6%. The majority (96.2%) of patients underwent lobectomy, while 3.8% received total pneumonectomy. Patients receiving PORT were more likely to be older (58.73%), of white ethnicity (78.96%), and diagnosed with adenocarcinoma (65.09%). In contrast, those who did not receive PORT were more likely to be female (55.13%) (Table 1).

**Table 1:** Description of the study population.

Variable	Total	PORT	No PORT
	*N* (%)	*N* (%)	*N* (%)
Age			
65–69 years	556 (37.8%)	253 (41.27%)	303 (35.31%)
70–74 years	915 (62.2%)	360 (58.73%)	555 (64.69%)
Sex			
Male	639 (43.4%)	254 (41.44%)	385 (44.87%)
Female	832 (56.6%)	359 (58.56%)	473 (55.13%)
Race			
White	1,158 (78.7%)	484 (78.96%)	674 (78.55%)
Black	138 (9.4%)	51 (8.32%)	87 (10.14%)
Other	175 (11.9%)	78 (12.72%)	97 (11.31%)
Year_of_diagnosis			
2010–2014	2 (0.1%)	0 (0.00%)	2 (0.23%)
2015–2019	1,469 (99.9%)	613 (100.00%)	856 (99.77%)
Primary_site			
Upper lobe	803 (54.6%)	352 (57.42%)	451 (52.56%)
Middle lobe	75 (5.1%)	35 (5.71%)	40 (4.66%)
Lower lobe	555 (37.7%)	213 (34.75%)	342 (39.86%)
Main bronchus	9 (0.6%)	1 (0.16%)	8 (0.93%)
Other	29 (2.0%)	12 (1.96%)	17 (1.98%)
Grade			
I	114 (7.7%)	39 (6.36%)	75 (8.74%)
II	715 (48.6%)	302 (49.27%)	413 (48.14%)
III	630 (42.8%)	264 (43.07%)	366 (42.66%)
IV	12 (0.8%)	8 (1.31%)	4 (0.47%)
Histology			
SCC	227 (15.4%)	81 (13.21%)	146 (17.02%)
ADC	908 (61.7%)	399 (65.09%)	509 (59.32%)
ADSC	33 (2.2%)	10 (1.63%)	23 (2.68%)
Large-cell carcinoma	3 (0.2%)	2 (0.33%)	1 (0.12%)
Other	300 (20.4%)	121 (19.74%)	179 (20.86%)
T_stage			
T1	571 (38.8%)	238 (38.83%)	333 (38.81%)
T2	900 (61.2%)	375 (61.17%)	525 (61.19%)
Operation type			
Lobectomy	1,415 (96.2%)	598 (97.55%)	817 (95.22%)
Pneumonectomy	56 (3.8%)	15 (2.45%)	41 (4.78%)

SCC, squamous cell carcinoma; ADC, adenocarcinoma; ADSC, adipose-derived stem cells; PORT, postoperative radiotherapy.

### 3.2 Comparison of Kaplan-Meier survival curves

**Figure 1:**
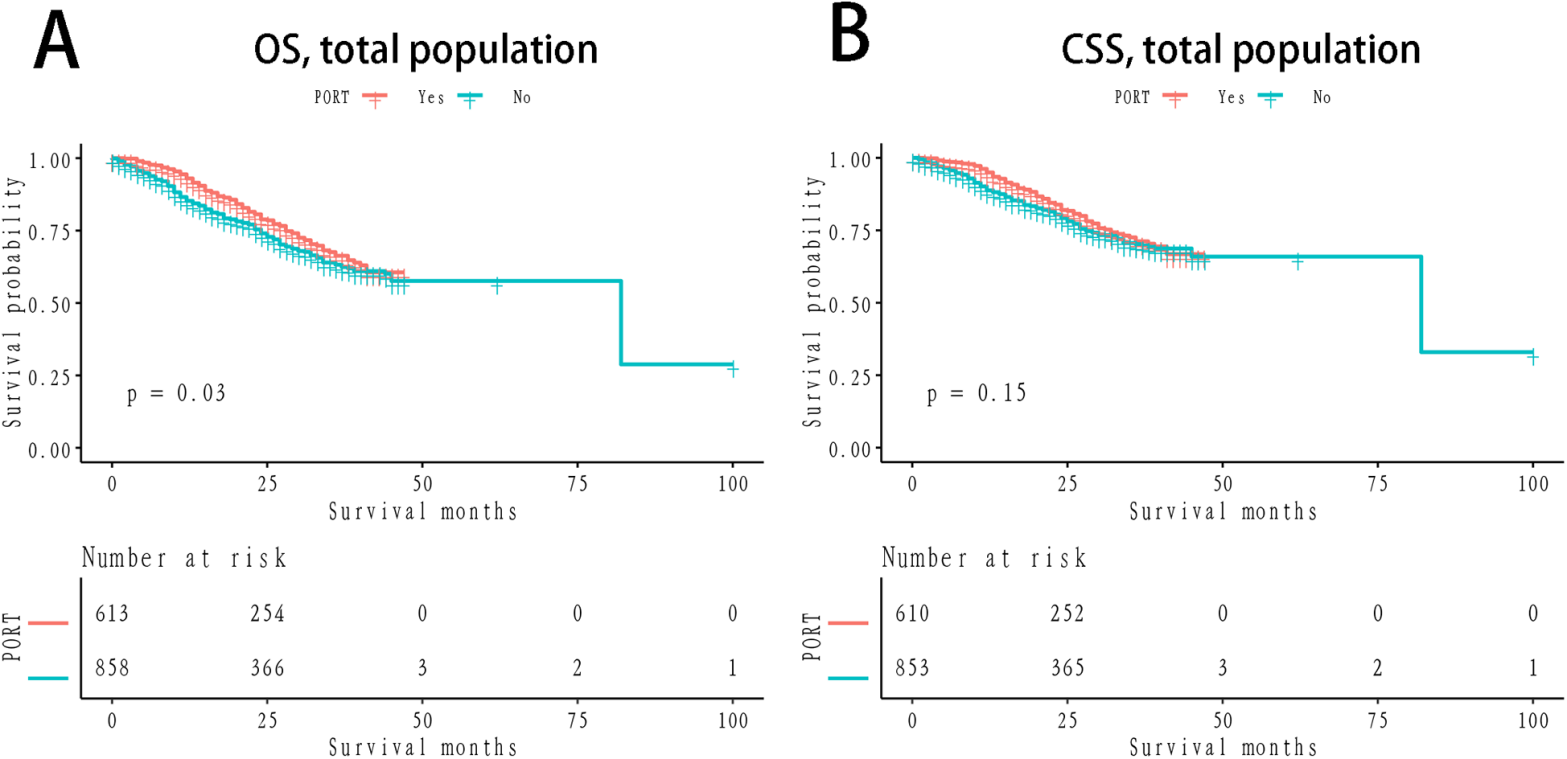
Km survival curves grouped by PORT **(A)** OS, stratified by NLN; **(B)** CSS; OS: verall survival; CSS, cancer-specific survival; KM, Kaplan-Meier; PORT, postoperative radiotherapy.

**Figure 2:**
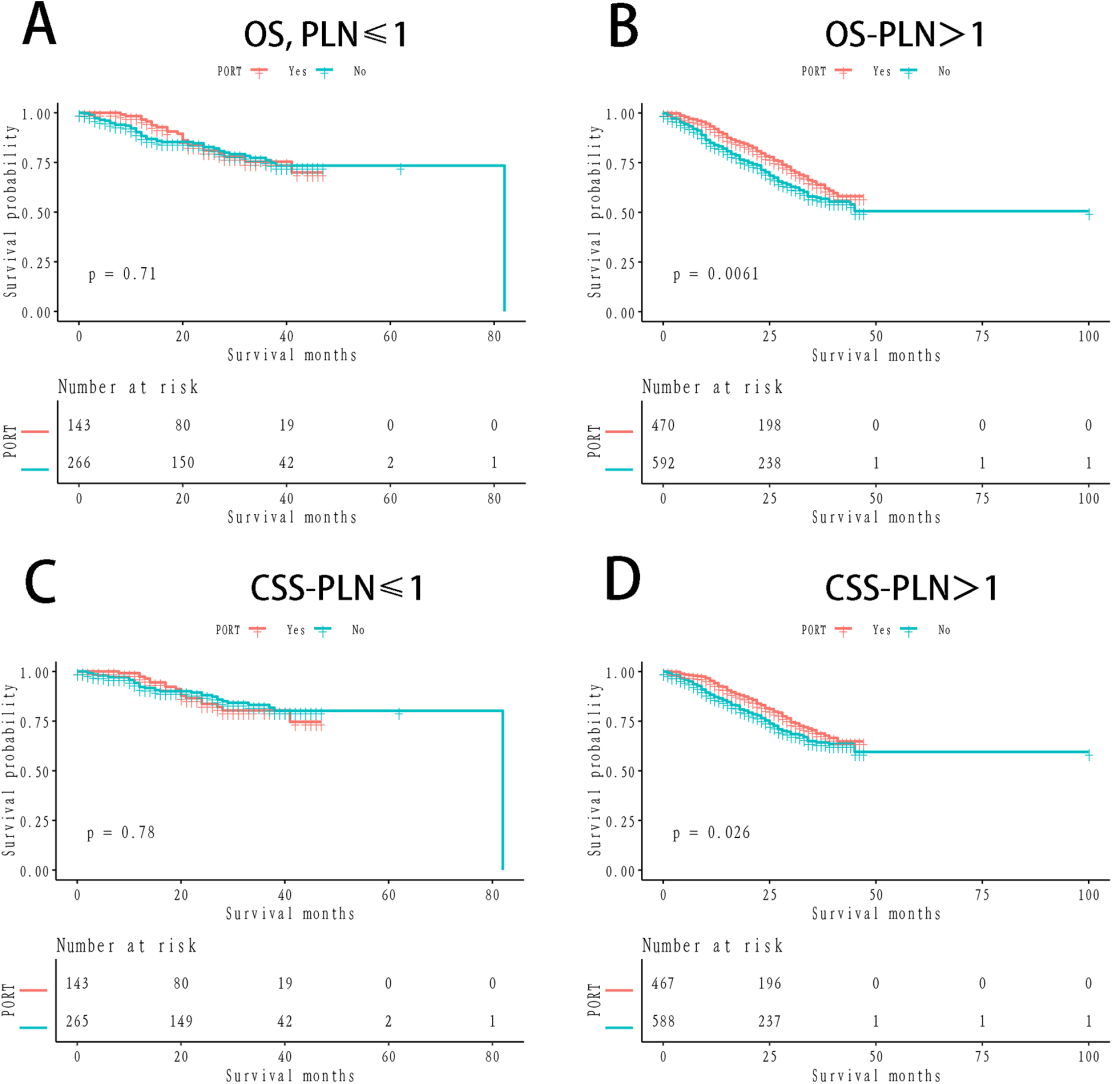
Km survival curves stratified by positive lymph nodes counts **(A)** OS, people with negative lymph nodes ≤1; **(B)** OS, people with negative lymph nodes >1; **(C)** CSS, people with negative lymph nodes ≤1; **(D)** CSS, people with negative lymph nodes >1.

The 1- and 3-year OS rates in the PORT group were 89.96% and 68.49%, respectively, while the non-PORT group exhibited 1- and 3-year OS rates of 87.44% and 61.88%, respectively. The difference between the two groups was statistically significant (*P* = 0.03; Figure 1A). In terms of CSS, the 1- and 3-year rates in the PORT group were 92.22% and 73.24%, respectively, compared to 90.77% and 68.91% in the non-PORT group. This difference did not reach statistical significance (*P* = 0.15; Figure 1B). In the stratified Kaplan-Meier analysis, when OS was the outcome measure, there was no significant survival difference between the PORT and non-PORT groups in patients with a PLN count of ≤1 (*P* = 0.71; Figure 2A). However, for patients with PLN >1, the difference in OS was statistically significant (*P* = 0.0016; Figure 2B). A similar trend was observed for CSS: in patients with PLN ≤1, the difference was not significant (*P* = 0.78; Figure 2C), whereas in patients with PLN >1, the survival difference was statistically significant (*P* = 0.026; Figure 2D).

### 3.3 Univariate and multivariate analyses

Univariate analysis revealed that variables such as age, sex, primary tumor site, histological subtype, T stage, PORT, and PLN count were significantly associated with OS and CSS (Table 2). In the fully adjusted model, we accounted for potential confounders, including age, sex, primary tumor site, histology, T stage, PORT, and PLN count.

**Table 2:** Univariate analysis.

Variables	All cause of death	Special deaths from lung cancer
	HR (95% CI) *P*	HR (95% CI) *P*
Age		
65–69 years	1	1
70–74 years	1.5 (1.2, 1.9) <0.001	1.6 (1.2, 2.1) <0.001
Sex		
Male	1	1
Female	0.7 (0.5, 0.8) <0.001	0.7 (0.5, 0.8) <0.001
Race		
White	1	1
Black	1.1 (0.7, 1.5) 0.754	1.0 (0.7, 1.5) 0.916
Other	0.7 (0.5, 1.0) 0.089	0.7 (0.5, 1.1) 0.134
Year_of_diagnosis		
2010–2014	1	1
2015–2019	1.7 (0.2, 13.9) 0.630	1.4 (0.2, 12.1) 0.760
Primary_site		
Upper lobe	1	1
Middle lobe	0.9 (0.5, 1.5) 0.681	1.0 (0.6, 1.8) 0.968
Lower lobe	1.4 (1.1, 1.7) 0.002	1.4 (1.1, 1.8) 0.006
Main bronchus	0.4 (0.1, 3.1) 0.413	0.0 (0.0, Inf) 0.991
Other	0.6 (0.2, 1.6) 0.304	0.8 (0.3, 2.1) 0.607
Grade		
I	1	1
II	1.3 (0.8, 2.1) 0.238	1.3 (0.8, 2.1) 0.375
III	1.5 (1.0, 2.4) 0.072	1.5 (0.9, 2.6) 0.113
IV	2.4 (1.0, 6.0) 0.057	2.5 (0.9, 6.8) 0.075
Histology		
SCC	1	1
ADC	0.7 (0.5, 0.9) 0.002	0.7 (0.5, 1.0) 0.036
ADSC	0.5 (0.2, 1.1) 0.085	0.5 (0.2, 1.2) 0.137
Large-cell carcinoma	0.4 (0.0, 3.4) 0.407	0.5 (0.1, 4.6) 0.556
Other	0.6 (0.4, 0.8) <0.001	0.6 (0.4, 0.9) 0.010
T_stage		
T1	1	1
T2	1.3 (1.0, 1.6) 0.018	1.5 (1.1, 1.9) 0.004
Operation type		
Lobectomy	1	1
Pneumonectomy	1.5 (1.0, 2.4) 0.063	1.3 (0.7, 2.3) 0.354
PORT		
Yes	1	1
No	1.3 (1.0, 1.6) 0.031	1.2 (0.9, 1.5) 0.149
Regional_nodes_positive categorical		
≤1	1	1
>1	1.7 (1.3, 2.2) <0.001	1.8 (1.4, 2.5) <0.001

SCC, squamous cell carcinoma; ADC, adenocarcinoma; ADSC, adipose-derived stem cells; PORT, postoperative radiotherapy.

Table 3 displays the results of the multivariate analysis. When OS was the primary endpoint, among patients with PLN ≤1, the adjusted all-cause mortality rate was 1.08 times higher in those who did not receive PORT compared to those who did (HR = 1.08, 95% CI = 0.64–1.82, *P* = 0.7790), though this result was not statistically significant. In the group with PLN >1, the adjusted all-cause mortality rate was 1.32 times higher in the non-PORT group than in the PORT group (HR = 1.32, 95% CI = 1.04–1.68, *P* = 0.0244), a statistically significant finding.

**Table 3:** Multifactorial analysis.

PLN	PORT	All cause of death		
		Non-adjusted	Adjust I	Adjust II
		HR (95% CI) *P*	HR (95% CI) *P*	HR (95% CI) *P*
PLN ≤1	PORT	1	1	1
	No PORT	1.10 (0.66, 1.83) 0.7086	1.11 (0.67, 1.85) 0.6864	1.08 (0.64, 1.82) 0.7790
PLN >1	PORT	1	1	1
	No PORT	1.39 (1.10, 1.76) 0.0067	1.35 (1.06, 1.71) 0.0144	1.32 (1.04, 1.68) 0.0244
PLN	PORT	Special deaths from lung cancer		
		Non-adjusted	Adjust I	Adjust II
		HR (95% CI) *P*	HR (95% CI) *P*	HR (95% CI) *P*
PLN ≤1	PORT	1	1	1
	No PORT	0.92 (0.52, 1.64) 0.7792	0.94 (0.53, 1.69) 0.8457	0.93 (0.51, 1.70) 0.8240
PLN >1	PORT	1	1	1
	No PORT	1.35 (1.03, 1.76) 0.0275	1.31 (1.00, 1.71) 0.0473	1.30 (0.99, 1.70) 0.0592

Non-adjusted model adjust for: none; adjust I model adjust for: age; sex; adjust II model adjust for: age; sex; primary_site; histology; T_stage; HR, hazard ratio; PORT, postoperative radiotherapy.

When CSS was used as the outcome measure, similar results were observed. In patients with PLN ≤1, the adjusted all-cause mortality rate was 0.93 times higher in those without PORT compared to those with PORT (HR = 0.93, 95% CI = 0.51–1.70, *P* = 0.8240), a result that was not statistically significant. Among patients with PLN >1, the adjusted all-cause mortality rate was 1.30 times higher in those without PORT compared to those with PORT (HR = 1.32, 95% CI = 0.99–1.70, *P* = 0.0592), though this difference was not statistically significant.

## 4 Discussion

This study demonstrated an improvement in OS rates among patients with completely resected pN2, stage IIIA NSCLC treated with PORT compared to those not receiving PORT. This improvement was non-significant in patients with a PLN count of ≤1, but statistically significant in those with PLN counts >1. Patients with completely resected NSCLC and pN2 disease represent a highly heterogeneous cohort, exhibiting complex and variable treatment strategies, with survival rates ranging from 7% to 36% (8–10). With the increasing adoption of advanced radiotherapy techniques and optimized dosing regimens, many prior retrospective studies suggest that PORT may improve survival outcomes in this population (11, 12).

The role of PORT in NSCLC has been evaluated for decades, yet despite several trials and meta-analyses, its clinical benefit remains a subject of ongoing debate. The heterogeneity of results across different stages of the disease adds to this controversy. For instance, a study by Lafitte et al. (13), which focused on pN0 patients, found no significant difference in overall survival or local control between surgery combined with PORT and surgery alone. Another study by Trodella et al. similarly evaluated PORT in pN0 patients (14), using 28 fractions of 1.8 Gy to a total dose of 50.4 Gy. Although the initial results, published in 2002, indicated a positive trend for PORT in 5-year overall survival (67% vs. 58%, *P* = 0.046), this was not confirmed upon updating the data for reanalysis in the PORT meta-analysis (15). The latter showed a detrimental effect of PORT in patients with completely resected pN0 and pN1 NSCLC (16, 17). In the ANITA trial, which compared adjuvant chemotherapy to observation in patients with completely resected stage IB to stage IIIA NSCLC, there was a significant 8.6% improvement in 5-year OS in the chemotherapy arm (17, 18). However, *post hoc* analysis of the ANITA study also demonstrated a negative impact of PORT in pN0–1 patients, leading to the current recommendation against PORT in this subgroup.

Although better locoregional control with PORT has been shown in the Lung ART and PORT-C trials, this has not yet resulted in an overall survival improvement. In the study by Dautzenberg et al. (19), which remains the largest PORT meta-analysis to date, the authors reported an adverse impact of PORT on survival, with a 5-year overall survival rate of 30% in the PORT cohort compared to 43% in the control group (*P* = 0.002). Nevertheless, in patients with stage N2 disease, PORT has been shown to reduce local recurrence rates. The disproportionate number of deaths in patients treated with PORT is largely attributable to the high incidence of cardiac and respiratory complications, such as cardiopulmonary failure, radiation pneumonitis, and massive hemoptysis. These outcomes have been influenced by outdated radiotherapy techniques and the associated high morbidity. In light of advancements in radiotherapy, the role of PORT in resected pN2 NSCLC warrants reevaluation. Recent studies have highlighted the significant benefits of radiotherapy (20), demonstrating lower morbidity with modern radiotherapy techniques compared to cobalt units, and suggesting a substantial reduction in the risk of cardiac mortality (21).

Studies on the N2 population, however, have shown contradictory findings. A wide range of clinicopathological characteristics, including lymph node (LN) involvement (number of afflicted stations or areas), LN volume (big vs. non-large), initial tumour size, and histological subtype, characterise the extremely varied group of patients with stage III pN2 NSCLC. Local recurrence rates and prognosis have been linked to the volume and extent of N2 illness (22–25). Patients with substantial LN involvement (several N2 metastases or stations) showed increased OS with PORT, according to a recent meta-analysis by Liu et al. (22), whereas patients with single-station N2 involvement showed no benefit.

A randomized study by the Lung Cancer Study Group (LCSG) included 230 patients with resected stage II or III squamous cell carcinoma and showed that PORT significantly prolonged disease-free survival (DFS) in patients with stage N2 (22). Similarly, the Medical Research Council (MRC) trial, which had a design akin to the LCSG study, demonstrated a trend toward improved overall survival in N2 patients. A plausible explanation is that PORT may be detrimental in early-stage patients (stage I or II) but confers a survival advantage in stage N2 patients due to the higher risk of recurrence in this subgroup.

A Japanese retrospective study further explored the impact of PORT based on the number of LN stations involved. Although PORT did not significantly affect overall survival, it markedly improved DFS by reducing local recurrence in patients with multi-station N2 involvement. Consistent with these findings, Urban et al. (26). analyzed 11,324 patients from the SEER database and observed that PORT conferred a survival benefit in patients with pN2 disease when the lymph node ratio (positive lymph nodes/total resected lymph nodes) was at least 50%.

Among the known prognostic factors, LN status remains the most significant predictor of outcome in NSCLC patients. The 9th edition of the TNM classification continues to define lymph node staging based on the anatomical location of positive LNs, as in the previous 8th edition. However, in breast, gastric, and colorectal cancers, the TNM classification has evolved to incorporate the number of metastatic lymph nodes (MLNs) in staging. In NSCLC, the number of positive LNs is strongly correlated with prognosis, though this is contingent upon the adequacy of LN sampling at the time of surgery. Numerous studies have suggested that the count of positive LNs is a more robust prognostic indicator and could serve as an alternative to traditional pathologic N-staging (27, 28). It has been demonstrated that an increased number of positive LNs correlates with poorer survival outcomes, a trend more pronounced in pN2 patients compared to those with pN1 disease (29, 30). Multivariate analyses have consistently identified the number of metastatic LNs as a significant predictor of OS and DFS. In particular, among patients with ≥2 N2 lymph node metastases, PORT improved OS compared to those without PORT, while no significant difference was observed in patients with a single N2 metastasis. The findings of this study align with these results, suggesting that PORT may improve survival in patients with multiple N2 lymph node metastases.

However, several limitations of this study should be acknowledged. First, this was a retrospective analysis of data from the SEER database, rather than a prospective randomized controlled trial. Second, key prognostic variables such as smoking history, type of surgery, N2 station, and number of positive lymph nodes were not included in the analysis. This study is limited by the lack of detailed information on specific lymph node stations in the SEER database. As a result, we were unable to assess whether involvement of certain stations, such as station 7 (subcarinal lymph nodes), was associated with worse outcomes. Existing literature suggests that station 7 involvement may correlate with poorer prognosis due to its central location and potential for extensive tumor spread (31). Future studies incorporating more granular clinical data, including lymph node station information, are needed to address this limitation and further elucidate its impact on survival outcomes. While our study stratified patients based on the number of positive lymph nodes (PLN ≤1 vs. PLN >1), the SEER database does not provide detailed information on the number of involved lymph node stations (e.g., 2, 3, or 4 stations). This limits our ability to directly assess the outcomes of patients with varying degrees of lymph node station involvement. However, our findings suggest that patients with PLN >1 may derive greater survival benefits from PORT, indicating that increasing lymph node involvement likely amplifies the therapeutic effect of postoperative radiotherapy. Future studies incorporating station-level lymph node data are needed to validate and expand upon these findings. Additionally, adjuvant treatments such as chemotherapy, targeted therapy, and endocrine therapy were not considered. In addition, due to the inherent limitations of the SEER database, specific details regarding the dosing and fractionation of PORT, as well as the effects of other postoperative treatments, were unavailable. Lastly, the distinction between preoperatively known N2 and intraoperative incidental N2 is an important factor that could influence surgical and adjuvant treatment decisions. Unfortunately, the SEER database does not provide sufficient detail to make this distinction, which may be a limitation of our study. Additionally, the database lacks information on the number and location of involved lymph node stations (e.g., single-station vs. multi-station N2), which is known to significantly impact prognosis and treatment strategies. Future studies incorporating more detailed clinical and pathological data are warranted to address these gaps and provide further insights into the optimal management of stage IIIA pN2 NSCLC.

## 5 Conclusion

In conclusion, this study underscores the potential of PORT to enhance overall survival in patients with resectable pN2, stage IIIA non-small cell lung cancer, particularly in those with a positive lymph node count exceeding one. These findings highlight the need for further prospective studies to substantiate and expand upon these observations.

## Data Availability

Publicly available datasets were analyzed in this study. This data can be found here: https://seer.cancer.gov/.
